# Characteristics of multi-absorption bands in near IR based on a 1D photonic crystal comprising two composite metamaterials

**DOI:** 10.1038/s41598-024-51229-x

**Published:** 2024-01-11

**Authors:** Mai Medhat, Ahmed Mehaney, M. Al-Dossari, Arafa H. Aly, Hussein A. Elsayed

**Affiliations:** 1 TH-PPM Group, Physics Department, Faculty of Science, Beni-Suef University, Beni-Suef, 62512 Egypt; 2 Department of Physics, Faculty of Science, King Khalid University, Abha, 62529 Saudi Arabia; 3 Department of Physics, College of Science, University of Ha’il, Ha’il, P.O. Box 2440 Saudi Arabia

**Keywords:** Materials science, Optics and photonics, Physics

## Abstract

The Matlab program has been utilized in this study to examine the absorption spectral properties of a one-dimensional photonic crystal (1DPCs) comprising two composite metamaterials through near IR wavelengths. The composite metamaterials are designed from Ag of a gyroidal geometry (layer A) and hyperbolic metamaterial (layer B). Therefore, the introduced design is labeled as ${[A{B}^{n}]}^{m}$ with n and m to define the periodicity of the hyperbolic metamaterial and the whole structure, respectively. The numerical findings have been introduced in the vicinity of the effective medium theory, transfer matrix method and the Drude model as well. In this regard, the numerical results demonstrate the appearance of some spectral absorption bands ranging from 0.7 µm to 3 µm for both TM and TE polarizations. Additionally, these bands are almost insensitive to the changes in the angle of incidence. Interestingly, we have considered the role played by some parameters such as the permittivities and thicknesses of both layers on the introduced absorption bands. Finally, we believe that the investigated results could be promising through many applications such as wavelength selective absorbers, solar energy, and smart windows as well.

## Introduction

Metamaterials (MMs) have attracted a lot of attention lately because of their wide range of applications^1–4^. These include spatial filters, perfect lenses, optical absorbers, chemical and biological sensors, and reflectors. MMs as manmade nanostructures with exact geometry and shape are characterized by a negative indices of refraction and atypical optical response as well^5,6^), MMs are classified as a novel type of designated left-hand material that provide a promising optical response during the interaction with the electromagnetic waves (EMWs)^7–9^. Meanwhile, the enthusiasm of researchers increases for employing and implementing features of MMs specifically when embedded in photonic crystals (PCs) designs. PCs are hetero-nano or submicron periodic structures that consist of different types of materials^7,10–13^. Interestingly, different types of materials including nanocomposites, dielectrics, semiconductors, metals, 2D materials and superconductors are widely considered through the design and fabrication of PC structures^14,15^. In PCs, the prohibited propagation of EMWs through a particular frequency range is expected across the formation of some optical stop frequency bands named photonic band gaps (PBGs)^16,17^. Such stop bands are very sensitive to incident angle due to constructive or destructive interferences of based on Bragg's law in 1DPCs^14–17^. Additionally, at any angle of incidence, the frequency or wavelength ranges of PBGs cross to provide an omnidirectional PBG. Depending on how they are sensitive to even small changes in refractive index, 1DPCs have intriguing optical features that make them an attractive tool for improving optical systems. Consequently, the majority of PC applications are in optical biosensors and physical applications^18–20^. For example, optical fibers, logic gates, solar cells, optical mirrors, absorbers, and optical communications have all been operated by PCs^11,21^.

Nowadays, immersing optical MMs in PC design became a milestone in electromagnetic society^22^. Recently, some new types of MMs named hyperbolic metamaterial (HMM) and Gyroidal metamaterial (GMM) configurations received some significant attention^23–26^. GMM is a novel design of MMs that received increasing interest through theoretical studies and experimental techniques^23^. Interestingly, GMM could be designed in a tri-helical geometry which is comprised of a superfine layer of metal in a three-dimensional configuration based on a dielectric hosting medium^24–26^. Moreover, GMM has a zero-average curvature at all points in its tri-helix geometry owing to its negative permittivity of a real part^27–29^. Furthermore, experimental investigations demonstrated the ease of manufacturing of such periodically immersed metallic structures in a dielectric substance. These features make GMM a pioneering nanostructure in numerous applicable fields, such as switches, thermal emission, and sensors or detectors^30,31^.

However, HMMs are a novel class of isotropic metamaterial, also referred to as uniaxial medium and anisotropic MM^32,33^. The unusual dielectric is an open hyperboloid of iso-frequency surface, and HMM exhibits unique features in the form of magnetic and electric components with opposite signs^34,35^. Also, the dissipative loss of anisotropy MM naturally reduces than in a metallic structure. One key point from HMM characteristics achieves negative refraction so that recalled indefinite MMs^36,37^. Meanwhile, indefinite metamaterials could be represented by periodic nanocomposite of dielectric-metallic layers^38,39^. It's interesting to note that hyperbolic media interacts with light propagation through more flexibly regulated design; they can be employed as sub-wavelength light concentrators in nanolithography, super lenses, and light absorbers in photonics and photovoltaics^35,40,41^.

In this work, we aim to develop a theoretical investigation to the absorption characteristics of a 1D PCs comprising two composite MMs. Here, our suggested design is configured as, [AB^n^]^m^ with A to define the GMM and B to describe the HMM. The GMM is designed from Ag through a hosting dielectric medium of a gyroidal geometry. In contrast, the HMM is a composite layer of (Ag) and indium arsenide (InAs) with a specified volume fraction. The numerical findings are essentially introduced based on some theoretical tools like the effective medium theory, transfer matrix method and Drude model as well. The investigated results demonstrate the formation of wide spectral absorption bands through the near IR wavelengths. The role of some parameters such as the angle of incidence, mode of polarization, layers’ thicknesses and the permittivities of both GMM and HMM are extensively discussed. With the explanation of these previous factors, our structure also shows the benefits and characteristic properties of using two different composites of MMs. Thus, our design can be used in many applications in the IR region of the electromagnetic waves spectrum owing to its in-sensitivity angle property. In addition, the Tri-helix configuration of GMM and the matching or congener within its components lead to zero curvature and makes it the best choice for biological, chemical and medical sensors^9,42^. Furthermore, this structure attracts our attention for its absorption bands in different positions of IR spectrum range^43^. Therefore, we can employ these properties towards some energy applications such as perfect absorbers, an intermediate layer for solar cells, polarization selector, super lenses, and optical switches.

## Theoretical method and model design

**Figure 1:**
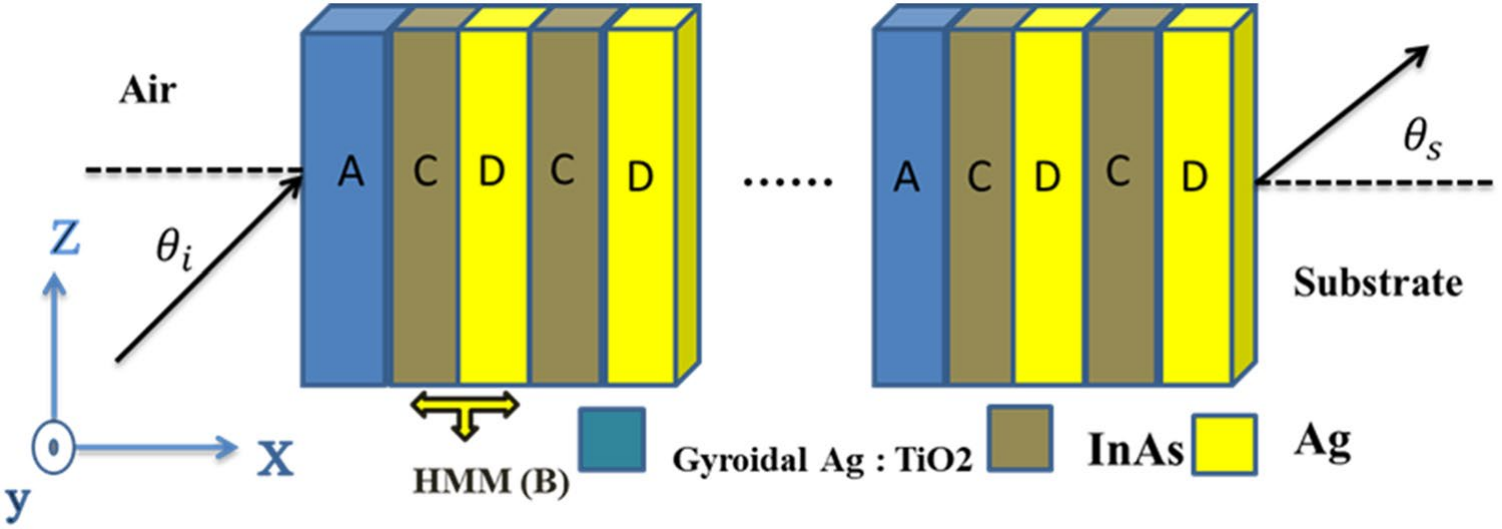
The schematic diagram of a 1D PCs composed of alternating GMM and HMM layers as a base of our design.

According to Fig. 1, the structure of a 1DPC is made up of two alternating layers of hyperbolic metamaterial HMM (layer B) and gyroidal metamaterial GMM (layer A). Therefore, the designed structure is labeled as is ${[A{B}^{n}]}^{m}$ with n and m to define the periodicity of the HMM layer and the whole structure, respectively. In this case, the superfine Ag metal is embedded in a gyroidal hosting medium of titanium dioxide (TiO_2_), to design layer A with thickness, d_A_ = 40 nm, and the refractive index, n_A_. In contrast, layer B is a composite layer of two different materials i. e., (CD). Here, layer C is designed from indium arsenide (InAs) and layer D is constructed from silver (Ag). Then, ${n}_{C},{n}_{D}$ describe the refractive indices of layers C and D whose thicknesses are ${d}_{C}=100 nm$, ${d}_{D}=40 nm$, respectively. This proposed design is constructed as a sandwich between air medium and glass substrate.

Consequently, the absorption spectra are theoretically determined by Bloch’s state and the well-known transfer matrix formulism which describes the interactions of EMWs with the proposed structure. For m number of unit cells, the total characteristic matrix for our candidate structure can be written as^44,45^:

1 $${M}_{t}={\left(\prod_{i=1}^{n}{M}_{i}\right)}^{m}=\left(\begin{array}{cc}{M}_{11}& {M}_{12}\\ {M}_{21}& {M}_{22}\end{array}\right)={( {M}_{A}{M}_{B})}^{m}$$

where, ${M}_{t},{M}_{A},{M}_{B}$ are the matrices of the whole structure, GMM, and HMM layers, respectively.

The interaction of the incident radiation within our proposed design can be described in the vicinity of the interaction through each layer *i* along the *x*-axis such as^46,47^:

2 $${M}_{i}=\left(\begin{array}{cc}{\text{cos}}({Q}_{i})& (-i/{\gamma }_{i}){\text{sin}}({Q}_{i})\\ {-i\gamma }_{i}{\text{sin}}({Q}_{i})& {\text{cos}}({Q}_{i})\end{array}\right)$$

In Eq. (2), For TE ${Q}_{i}$ = $\frac{2\pi {d}_{i}}{\lambda }{n}_{i}{\text{cos}}{\theta }_{i}$ and ${\gamma }_{i}={n}_{i}{\text{cos}}{\theta }_{i}$, then for TM polarization ${Q}_{i}$ = $\frac{2\pi {d}_{i}}{\lambda }{\text{cos}}{\theta }_{i}/{n}_{i}$,${\gamma }_{i}=\frac{{\text{cos}}{\theta }_{i}}{{n}_{i}}$. Now, the optical properties of layers a and B in the vicinity of their indices of refraction can be introduced based on the effective medium theory and Drude model as well^21,48^.

Initially, we find the optical constants of metals using Lorentz-Drude model^42^. The damped harmonic oscillator model is utilized to describe the optical characteristics of noble metals. A dielectric compound has been shown to act in the following form^49^:

3 $$\widehat{\varepsilon }\left(\omega \right)={\widehat{\varepsilon }}^{\prime}\left(\omega \right)+{\widehat{\varepsilon }}^{{\prime \prime}}\left(\omega \right)$$

In the previous equation, the first term ${\widehat{\varepsilon }}^{\prime}\left(\omega \right)$ shows the important role of the permittivity of our utilized metals, and also known as a free- electron or Drude model, which can be expressed as given^48,50,51^:

4 $${\varepsilon }_{Drude}={\widehat{\varepsilon }}^{\prime}\left(\omega \right)={\varepsilon }_{\infty }-\frac{{\left({\Omega }_{p}\right)}^{2}}{{\omega }^{2}+i\omega \Gamma }$$

whereas ${\varepsilon }_{\infty }$ is defined as a relative permittivity limit at high frequency, and ${\Omega }_{p}$ and $\Gamma$ are considered about the Plasma and damping frequency. Then, these constants for Gyroidal Ag have values ${\varepsilon }_{\infty }=5 {\text{ev}}{, \Omega }_{p}=9.01 {\text{ev}}$, and ћ$\Gamma =0.048\, {\text{ev}}$ with ћ as the reduced Planck’s constant^51–53^. Then, the refractive index of TiO_2_ $({n}_{T})$ as a hosting medium through the gyroidal layer is given as^54,55^:

5 $${n}_{T}=\sqrt{5.913+\frac{0.2441}{{\lambda }^{2}-0.0803} }$$

Next, the permittivity of GMM (${\varepsilon }_{A}$) is investigated as a function of some variables like, hosting medium refractive index ${n}_{T}$, metal permittivity ${\varepsilon }_{Drude}$, and geometrical constant of Gyroidal configuration such that^4,56–58^:

6 $${\varepsilon }_{A}= 1.193\left({\sqrt{2}-16\sqrt{2 }G}^{2}{\left(\frac{\pi \sqrt{-{\varepsilon }_{Drude}}}{2\sqrt{2} {n}_{T}}-1\right)}^{2}\right)$$

7 $${R}_{A}=0.29 {r}_{A}\sqrt{h} , {\lambda }_{A}=1.15 {r}_{A}\sqrt{1-(0.65{\text{ln}}(h)})$$

In Eq. (6), $G=({R}_{A}/{\lambda }_{A})^{2}$ is a fundamental term related to the geometrical constant with ${R}_{A}$ to denote the radius of the gyroidal helix which is known as a function of helix length ${r}_{A}$ and filling fraction of the used metal Ag ($h$). ${\lambda }_{A}$ is represented as an effective geometrical element for adjusting Gyroidal Ag configuration.

Then, we have mathematically investigated the effective medium theory to describe the permittivity of HMM^59^. Effective medium theory is comprised of a tensor formulism to compute the permittivity of HMM as following^41,60,61^:

8 $${\varepsilon }_{B}=\left[\begin{array}{ccc}{\varepsilon }_{Bx}& 0& 0\\ 0& {\varepsilon }_{Bx}& 0\\ 0& 0& {\varepsilon }_{BZ}\end{array}\right]$$

Here,${\varepsilon }_{B}$ refers to the HMM’s permittivity with ${\varepsilon }_{BZ},{\varepsilon }_{Bx}$ as the vertical and parallel components of its permittivity, respectively. Hence, these components are written in the next forms^35^:

9 $${\varepsilon }_{BX}={F\varepsilon }_{C}+\left(1-F\right){\varepsilon }_{D}$$

10 $${\varepsilon }_{BZ}= {\varepsilon }_{C}{\varepsilon }_{D}/{\varepsilon }_{D}F+{\varepsilon }_{C}(1-F)$$

So that, Eqs. (9), (10) illustrate the influence of indium arsenide permittivity (${\varepsilon }_{C}$) and silver permittivity (${\varepsilon }_{D}$) through $x$ and $z$ directions. The components of HMM ${{\varepsilon }_{Bx},\varepsilon }_{BZ}$ are represented as a function of filling fraction $F$, which can be variable with using the thicknesses of layers ${d}_{C},{d}_{D}$ as:

11 $$F=\frac{{d}_{C}}{{d}_{C}+{d}_{D}}$$

Additionally, the optical properties and responses of our design can be obtained from studying the coefficients of transmittance and reflectance, such that^21,62,63^:

12 $${t}_{1}=\frac{2{\gamma }_{0}}{\left({M}_{11}+{M}_{12}{\gamma }_{s}\right){\gamma }_{0}+({M}_{21}+{M}_{22}{\gamma }_{s})}$$

13 $${r}_{1}=\frac{\left({M}_{11}+{M}_{12}{\gamma }_{s}\right)2{\gamma }_{0}-{(M}_{21}+{M}_{22}{\gamma }_{s})}{\left({M}_{11}+{M}_{12}{\gamma }_{s}\right){\gamma }_{0}+({M}_{21}+{M}_{22}{\gamma }_{s})}$$

Finally, the transmittivity and reflectivity of the designed structure are given as^64,65^:

14 $$T=\frac{{\gamma }_{s}}{{\gamma }_{0}}\left|\left.{{t}_{1}}^{2}\right|\right.$$

15 $$R=\left|\left.{{r}_{1}}^{2}\right|\right.$$

Now, the absorption values of the designed PC structure can be investigated as:

16 $$A=1-\left(T+R\right)$$

## Results and discussion

**Figure 2:**
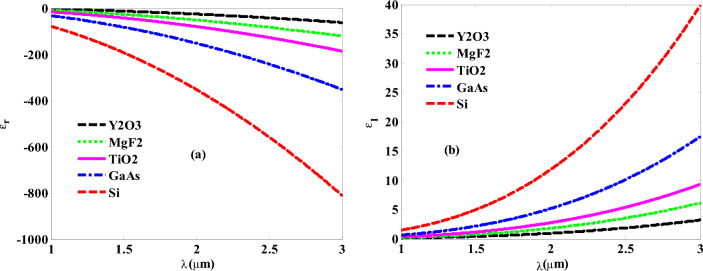
(**a,b**) The response of gyroidal layer’s permittivity under the effect of different hosting materials.

Now, we present in this section the numerical findings regarding the interaction of the incident EMWs with our designed structure. Firstly, we have introduced in Fig. 2 the impact of employing various hosting materials on the permittivity of GMM material. The figure shows that the inclusion of dielectric materials of different refractive indices leads to the increase in the values both real and imaginary parts of GMM’s permittivity. Moreover, the response becomes more dispersive with the increase in the wavelengths of the incident EMWs. By replacing yttrium oxide (Y_2_O_3_), with magnesium fluoride (MgF_2_), titanium oxide (TiO_2_), gallium arsenide (GaAs), and silicon (Si), the real part of GMM’s permittivity provides relatively large negative values that could reach to more than – 800 at a wavelength of 3 μm as shown in Fig. 2a. In contrast, the imaginary part received some increases with the replacing of Y_2_O_3_, by MgF_2_, TiO_2_, GaAs, and Si as shown in Fig. 2b.

**Figure 3:**
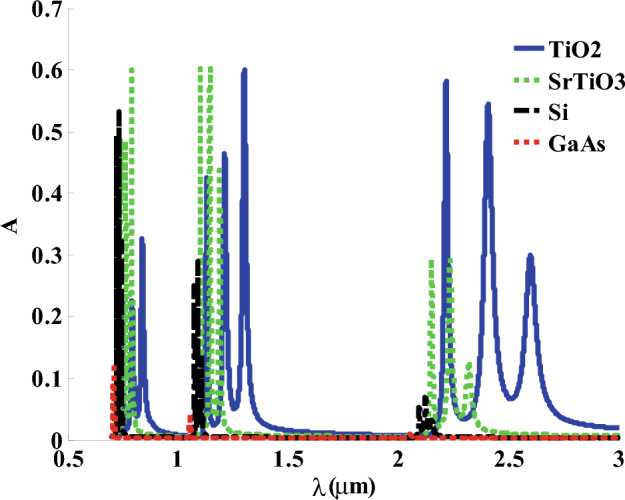
The characteristic absorption spectrum under the influence of different hosting materials.

Now, we have discussed in Fig. 3 the role of GMM’s permittivity on the absorption values of the designed PC structure. the figure indicates that the usage of a hosting material such as TiO_2_ and SrTiO_3_ with a relatively low refractive index leads to the appearance of some absorption bands through the wavelengths of interest. For materials with a relatively high refractive index like Si and GaAs^66^, the absorption bands are almost disappearing due to the increase in the structure reflectivity. In particular, at large indices of refraction for the hosting medium of GMM layer, the permittivity provides large negative values which could make the incident EMWs evanescent. Therefore, the role of the refractive index of the hosting material of GMM layer is crucial towards the formation of some absorption bands.

**Figure 4:**
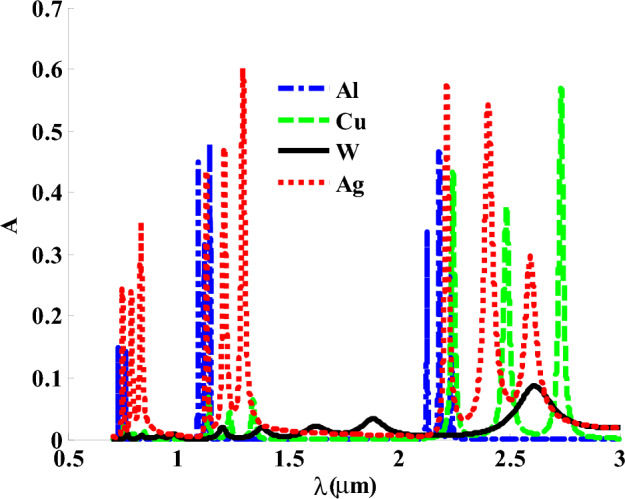
The effect of using different metals inside hosting material on the absorption ratio.

Here, we have produced our design with hosting material based on a variety of metals like both silver (Ag) and copper (Cu), Aluminum (Al), and Tungsten (W). These metals have some values of optical constant such as damping constant and plasmon frequency as inserted in Table 1. Therefore, the permittivities of these metals could be expressed based on Eq. (4) and the data listed in Table 1. Figure 4 shows some differences in the positions and intensities of the absorption bands as Ag is replaced with Al, W and Cu due to the difference in the values of the plasmon frequency and damping constant of these metals compared to Ag. In particular, the change in the values of plasmon frequency and damping constant could have a significant effect on the permittivity of the metal used and that of GMM as well. Thus, we believe that Ag represents the best choice compared to other metals due to its role in providing a suitable number of absorption bands besides their high absorption values as well through the considered wavelengths.

**Table 1:** Values of the plasma frequencies for the different metals^49,67^.

Metal	Plasma frequency ћ${\Omega }_{p}$ (eV)
Aluminum (Al)	14.98
Tungsten (W)	13.22
Copper (Cu)	10.83
Silver (Ag)	9.01

**Figure 5:**
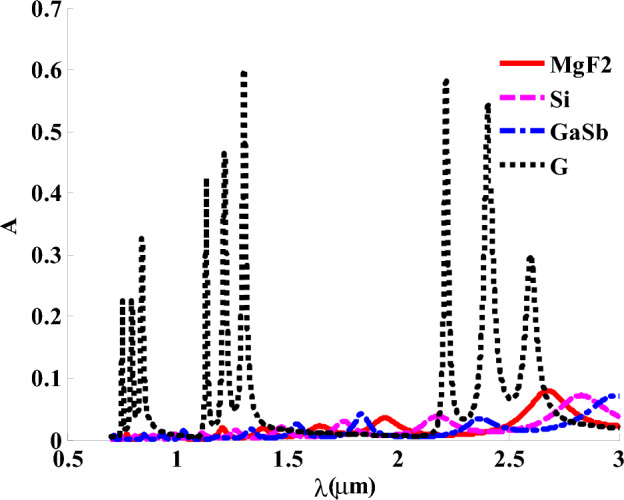
The response of absorption spectra under the effect of different materials instead of gyroidal metamaterial.

**Figure 6:**
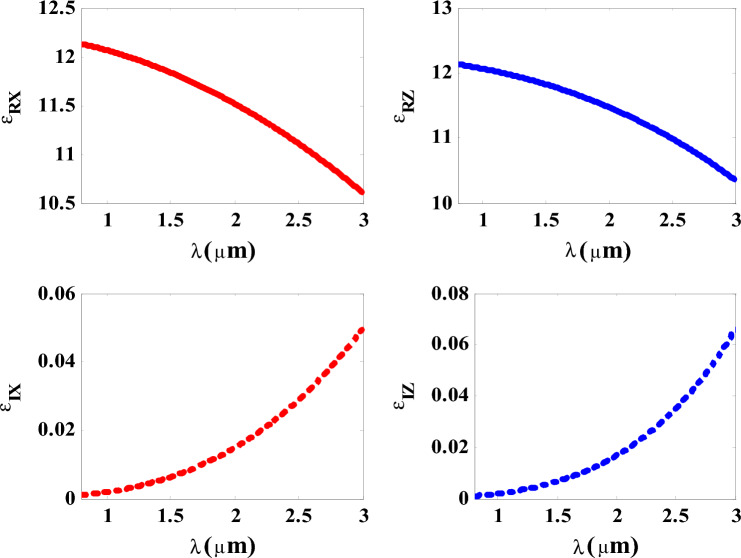
The effective relative permittivity of HMM in B multilayers as a function of the wavelength of the incident EMWs.

Figure 5 indicates that using a variety of materials like MgF_2_, Si, and Gallium antimonide (GaSb) cannot substitute the Gyroidal layer (G) with its unique metamaterial properties that are illustrated by the superb absorption value. Thus, these materials may be founded more suitable other applications far away about energy applications. Firstly, using MgF_2_ as a transparent crystalline material is commonly used in optical components like lenses, polarizers, prisms, and windows depending on studying its dispersion relation with incident wavelengths^68^. Secondly, GaSb is considered as a suitable component in infrared applications, but we found the absorption ratio does not exceed 0.1 of the spectrum. Then, using silicon element instead of Gyroidal metamaterial does not achieve the same value of absorption in the G state. Therefore, we consider G with its properties the best choice among these mentioned materials for our structure and the purposes of absorption applications through demonstrating three absorption bands with different ratios in both absorption and position in near IR region as shown in Fig. 5. in what follows, we present the role of the HMM on the absorption values of the designed structure. Firstly, we have introduced in Fig. 6 the permittivity’s values of HMM through the wavelengths of interest. Figure 6 thus depicts the real and imaginary components of the effective permittivity tensor of HMM against the wavelengths of the incident photons. Figure 6 indicates the values of the vertical and parallel components of HMM’s permittivity. Here, the real part of both vertical and parallel components of HMM’s permittivity provide positive values that received some decrements with the increase in the wavelength of the incident EMWs. In the other side, the imaginary part provide some small increases with the increase in the wavelength. However, the values of imaginary part for both vertical and parallel components are almost ineffective compared to those of real part.

**Figure 7:**
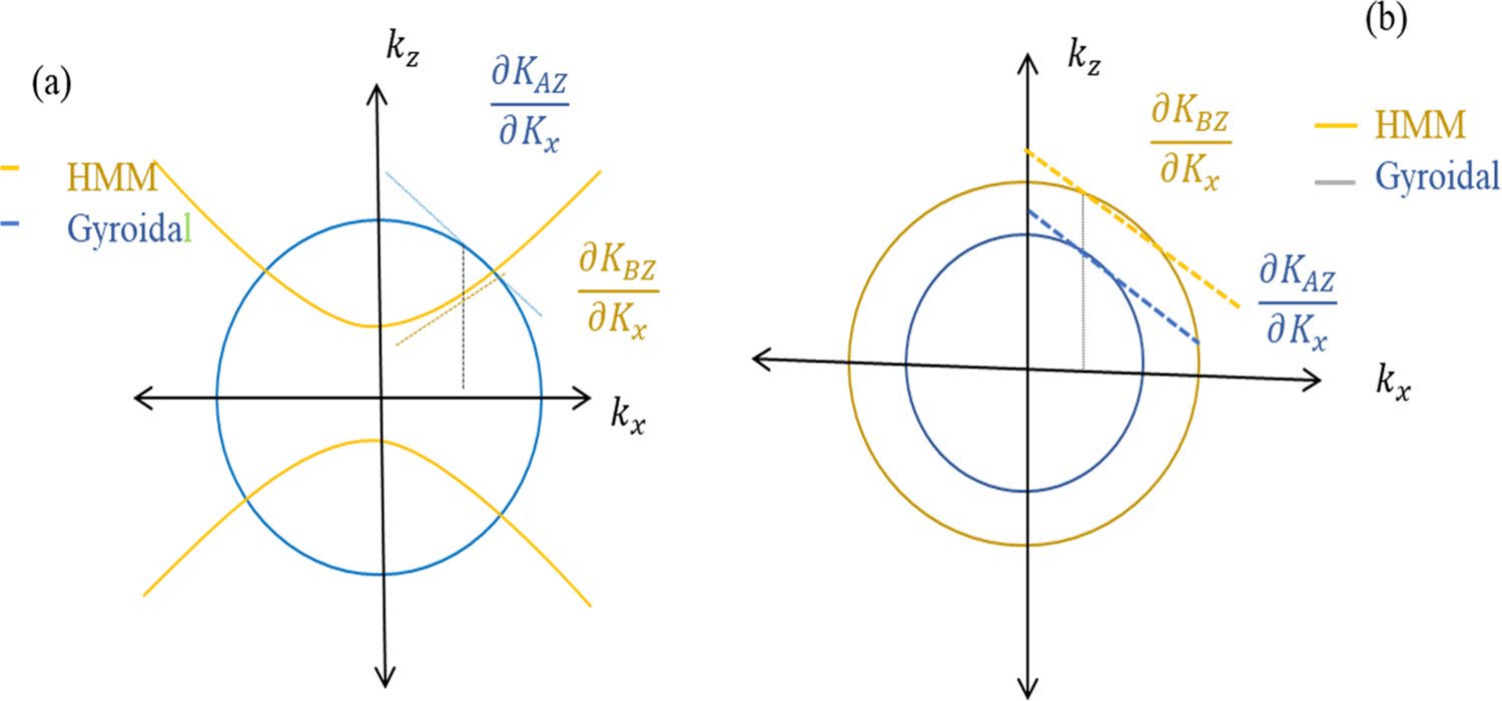
Iso-frequency curves of Gyroidal layer A and HMM layer B for both TM waves in (**a**) part and TE waves in (**b**) part in 1DPC structure.

Now, we turn our attention to discuss the iso-frequency curves of layers A and B. In particular, these curves indicate the role of HMM in providing an angle insensitive design. Figure 7 shows the response of *x* component ${K}_{x}$ of the wave vector that gradually increases with the increasing of incident angle. For TM modes in Fig. 7a, the iso –frequency curve of GMM (layer A) is circular however in HMM (layer B) this curve is hyperbolic due to the negative value of $\partial {K}_{AZ}/\partial {K}_{x}$ and positive value of $\partial {K}_{BZ}/\partial {K}_{x}$ , sequentially through the differentiation of the propagation phase $\phi$ as in the following given equation:

17 $$\frac{\partial \phi }{\partial {K}_{x}}=\frac{\partial {K}_{AZ}}{\partial {K}_{x}}{d}_{A}+\frac{\partial {K}_{BZ}}{\partial {K}_{x}}{d}_{B}$$

In contrast, the iso-frequency curves of both GMM and HMM are circular under TE modes as set in Fig. 7b. Thus, $\partial \phi/\partial {K}_{x}$ may be positive, zero or negative, when varying the thickness of GMM and HMM. It is worth mentioning that the total phase in a unit cell of our structure is still π according to Bragg condition as in the following equation^69^:

18 $$\phi ={\left|{K}_{AZ}{d}_{A}+{K}_{BZ}{d}_{B}\right|}_{{\lambda }_{Bragg}}=\pi$$

Consequently, the shift will be red-shifted, zero-shifted or blue- shifted in PBG^70,71^. Therefore, we declare the equations of iso-frequency curve of our structure parts under TM modes which are written as:

19 $$\frac{{{K}_{x}}^{2}}{{\varepsilon }_{A}}+\frac{{{K}_{AZ}}^{2}}{{\varepsilon }_{A}}={{K}_{0}}^{2} \quad \& \quad \frac{{{K}_{x}}^{2}}{{\varepsilon }_{B}}+\frac{{{K}_{BZ}}^{2}}{{\varepsilon }_{B}}={{K}_{0}}^{2}$$

where, the iso-frequency curve is mainly depending on the wave vector in vacuum ${K}_{0}$, and the wave vector of *x*- components.

**Figure 8:**
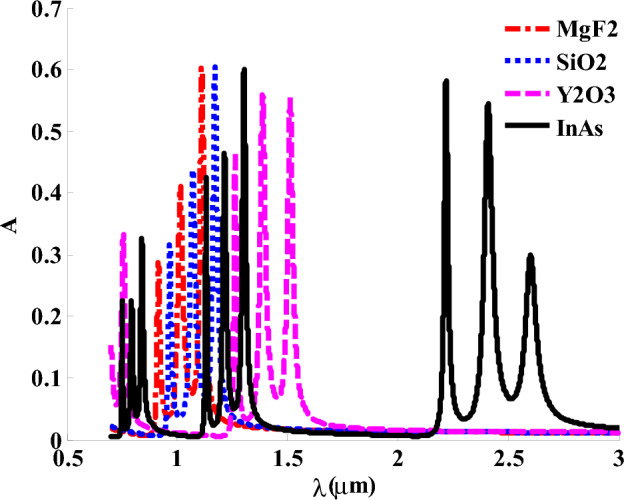
The characteristic absorption spectra under using different materials in HMM.

In Fig. 8, we have introduced the impact of layer C on the absorption value of the designed structure. The figure shows that the replacing of InAs with other materials like MgF_2_, SiO_2_ and Y_2_O_3_ could have a significant effect on the positions and intensities of the formed absorption bands. Here, the absorption bands shift downwards the short wavelength regions by replacing InAs with Y_2_O_3_, SiO_2_ and MgF_2_, respectively. This response is due to the decrease in the refractive index of layer C that could lead to some changes on the effective permittivity of HMM layer and the optical path length of the incident photons as well^72–74^. Thus, InAs represent the suitable choice inside HMM as it gives three suitable absorption band gaps extend from 0.74 to 0.9µm, 1.11 to 1.4µm,and 2.1 to 2.7µm, and is also known for its high electron mobility. In addition to that, InAs is a key material in many implementations such as infrared detectors, fabrication of quantum dot structures, and night vision systems.

**Figure 9:**
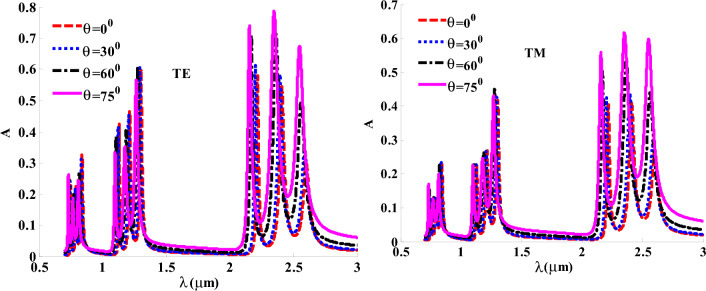
The absorption spectra for TE and TM polarization under different angles.

It is worth noting that Fig. 9 displays the absorption spectrum of our proposed design for TE and TM polarization at different angles of incidence. Meanwhile, we get symmetry in the absorption band gap position for TE and TM modes. This means that our structure achieves an angle-in-sensitive feature. In this regard, we also obtain three absorption bands at the same regions in TE and TM states of polarization with different values of absorption. Therefore, the widest absorption band which expands from 2 µm to 3 µm takes our attention as providing a high absorption ratio in the near IR region. In addition, the spectrum illustrates an increase in absorption ratio reaching 80% or 70% with increasing the angle of incidence.

**Figure 10:**
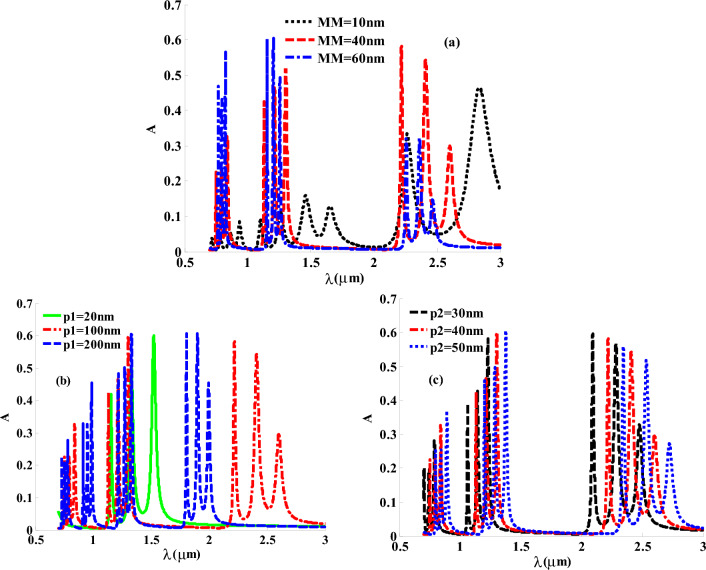
The absorption spectrum of the proposed 1D MMs PC design at different thicknesses of the constituent layers in (**a**) gyroidal metamaterial MM. (**b**) The first part of HMM $p1$ is noted in InAs. (**c**) The second part of HMM $p2$ composed of Ag metal.

Finally, we also discussed the role of thickness on our structure from each layer. Figure 10a declares that the effective role of GMM’s thickness on the absorption band ratio and position. At 10 nm thickness of GMM layer, the intensity of the absorption bands are relatively small especially at the short wavelength regions. For further increase in the thickness of GMM layer to 40 nm and 60 nm, the intensities of the absorption bands begin to increase with some decreases in their widths compared to the case of 10 nm. However, Fig. 10a demonstrates that 40 nm represents the optimum thickness of the GMM layer through our study. On the other hand, we pay more attention to the thickness of HMM and its effect on the absorption spectrum. Through decreasing the thickness of InAs from 100 to 20nm, we find one only one absorption band instead of three as at in the case of 100nm as shown in Fig. 10b. Nevertheless, Fig. 10b shows the shifting back towards shorter wavelengths as a result of increasing the thickness of layer C. In other words, we find the role of changing the second part of HMM thickness is represented in the displacement of band gap toward longer or shorter wavelengths as seen in Fig. 10c. The absorption in Near IR region is attributed to the optical localization, which caused by the interference effect at the interface of each layer.

## Experimental feasibility

Now, we briefly describe the experimental supports and facilitations of our designed 1DPC structure. Here, the 1DPC is presented based on two different MMs. In this context, the fabrication process passes by two main states like consisting of our design in two parts as labeled as ${[A{B}^{n}]}^{m}$. Meanwhile, the first part, which consists of silver with hosting material of TiO_2_ and the second part is established on InAs and ending by silver nano stack. In the front part of the proposed design, we practically deposit a hyperfine layer of silver through the gyroidal configuration of TiO_2_ dielectric hosting medium as a metamaterial composite. Furthermore, the fabrication of the first part can be completed by many techniques such as the thermal evaporation, RF sputtering method, electron-beam vacuum evaporator, and spin coating method. Moreover, the fabrication of gyroidal layer has been improved in 3D metallic structure as demonstrated in recent studies^29,75^. On the other side, the other part of our design can be manufactured as a film with unique characteristics in resistivity, optical and mechanical properties through chemical vapor deposition technique or physical spin-orbital coupling mechanism of waves^76–78^. As well as, the above-mentioned methodology, the fabrication facilitations of the 1D-PCs metamaterial can be experimentally applied easily from IR or UV and visible wavelength regions.

## Conclusion

In this work, the 1DPC structure is composed of the combination of two different MMs represented in a gyroidal configuration with HMM. The proposed design can achieve multi-absorption bands in IR region. Meanwhile, we have investigated the simulation work based on the Lorentz Drude model, transfer matrix formulism with effective medium theory, and Matlab software. Hence, the numerical findings demonstrated multi-absorption bands with high absorption ratio which are invariant in their position. These absorption bands mainly rely on the number of periodicity, which is configured as [${A{B}^{2}]}^{4}$ and other factors such as the used metal, type of hosting materials, and the components of HMM. Meanwhile, we hypothetically generated a near-infrared 1DPC with a zero-shifted PBG. Additionally, the multi-absorption bands extend from 0.74 µm: 0.9 µm to 1.1 µm: 1.4 µm and 2.1 µm to 2.7 µm. Furthermore, this structure reaches 80% or 70% of the absorption spectra, achieving a high absorption ratio. Our designed structure demonstrated an insensitive angle PBG in every band within the near IR spectrum. The fact that our structure is unique in that it can produce the same outcomes under TE and TM modes with larger incident angles. According to these results, we can consider our structure a good candidate for IR energy applications.

## Data Availability

The data that support the findings of this study are available from the corresponding author upon reason-able request.
